# IC-Tagging methodology applied to the expression of viral glycoproteins and the difficult-to-express membrane-bound IGRP autoantigen

**DOI:** 10.1038/s41598-018-34488-3

**Published:** 2018-11-02

**Authors:** Natalia Barreiro-Piñeiro, Irene Lostalé-Seijo, Rubén Varela-Calviño, Javier Benavente, José M. Martínez-Costas

**Affiliations:** 1 Group of Molecular Virology, Centro Singular de Investigación en Química Biolóxica e Materiais Moleculares (CiQUS), Departamento de Bioquímica e Bioloxía Molecular, Universidade de Santiago de Compostela, Santiago de Compostela, 15782 Spain; 2 Present Address: Centro Singular de Investigación en Química Biolóxica e Materiais Moleculares (CIQUS), Departamento de Química Orgánica, Universidade de Santiago de Compostela, Santiago de Compostela, 15782 Spain; 3 Departamento de Bioquímica e Bioloxía Molecular, Facultade de Farmacia, Universidade de Santiago de Compostela, Santiago de Compostela, 15782 Spain

## Abstract

We have previously developed a methodology to produce protein microspheres (MS) that can be loaded with proteins of interest in living cells through their C or N-terminal tagging with the so-called IC-Tag. The IC-Tagging method has many applications ranging from the production of immobilized enzymes for industrial use to the production of subunit vaccines due to its intrinsic adjuvancy. Here we show the adaptation of the IC-Tagging to work inside the endoplasmic reticulum and bacteria, allowing us to produce properly modified viral glycoproteins. Additionally, we were able to express the Islet-specific glucose-6-phosphatase catalytic subunit-related protein (IGRP), whose expression remained elusive to date possibly due to its toxicity when over-expressed. IGRP is an antigen of enormous pharmaceutical interest as it is specifically targeted during the autoimmune response taking place in both the Non-Obese Diabetic (NOD) mice and type 1 diabetes (T1D) patients leading to the destruction of insulin-producing beta cells.

## Introduction

**Figure 1:**
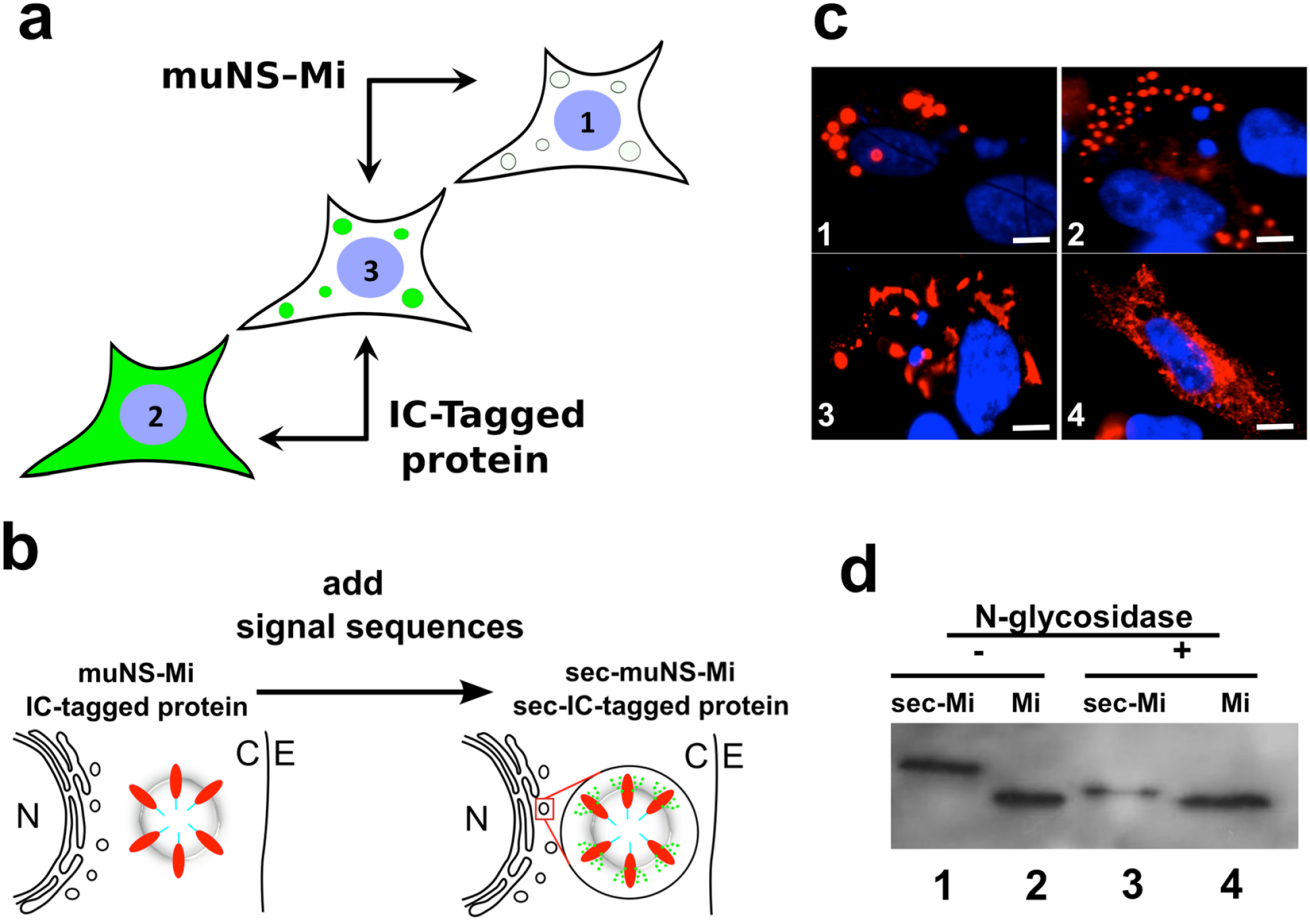
Formation of muNS-Mi microspheres inside the ER. (**a**) Schematic representation of the IC-Tagging methodology. muNS-Mi forms microspheres in the cytosol (1, white spheres). A protein tagged with the IC-Tag retains its normal location (2, a cytosolic protein seen green). When muNS-Mi and an IC-Tagged protein are expressed in the same cell, the IC-Tag re-localizes the IC-Tagged protein to muNS-Mi MS (3, green spheres). (**b**) The MS are cytosolic and can be loaded with cytosolic proteins (at the left). Our hypothesis: adding a signal peptide on the sequence of muNS-Mi will create a version of that protein (sec-muNS-Mi) that will form microspheres inside the ER, so they could be loaded with glycoproteins (on the right: the green spots represent sugar modifications). N-nucleus, C-cytosol, E-extracellular medium. (**c**) Immunofluorescence analysis of DF-1 cells transfected with plasmids expressing muNS-Mi (1) or sec-muNS-Mi (2, 3 and 4). Specific antibodies were used to detect muNS by indirect immunofluorescence (red) and nuclei were counterstained blue with DAPI. (**d**) Western-blot analysis performed with muNS-specific antibodies of extracts from DF-1 cells transfected with expression plasmids for muNS-Mi (Mi, 2 and 4) or sec-muNS-Mi (sec-Mi, 1 and 3) either before (−) or after treatment with N-glycosidase (+). Full length Western-blot is shown in Figure S7.

Many viruses build specific intracellular compartments for viral replication, called viroplasms, viral inclusion bodies or viral factories^1–3^. We have previously shown that avian reoviruses (ARV) use the viral non-structural protein muNS to form the scaffold of their irregular-shaped viroplasms^4^, while a truncated version of muNS, that we named muNS-Mi, forms quite regular protein spheres from 1 to 4 µm in diameter (microspheres or MS) when expressed alone inside any cell type^5^. In the same studies we have demonstrated that fusing a muNS-Mi domain called Intercoil (IC) to any protein of interest as a molecular tag, forces the re-localization of the IC-tagged protein to the muNS-Mi MS^6^ (Fig. 1a). This simple molecular tagging method has many applications, ranging from validation of protein-protein interactions inside the cytoplasm or nucleus of living cells^7^, to protein purification, including active enzymes^6^. We have also shown that proteins are properly folded inside the MS, where quaternary interactions occur and complex enzymatic reactions are allowed^8^.

Using the baculovirus/insect cell expression system, we can produce big amounts of MS that can be easily purified by simple mechanical methods due to their size and stability^6^. We have shown that bluetongue-virus (BTV) epitope-loaded MS serve as very effective subunit vaccines against BTV in IFNAR (−/−) mice, demonstrating their intrinsic adjuvant capacity, and opening the possibility of using the IC-tagging as a general method to produce safe and effective subunit vaccines^9^. The main advantages of our method, not fully shared by other methods used for production of subunit vaccines like virus-like particles (VLP) or chemically-synthesized nanoparticles are: i) particulate nature, a desired characteristic for inducing humoral and cellular immunity; ii) stability; iii) very simple, cheap and fast purification protocol; iv) there is no need to purify the epitopes previous to their coupling to a particle: the coupling is done by the cell; v) stabilization of the expressed epitopes; vi) they are fully biocompatible; vii) non-structural proteins, that are believed to participate in the development of a strong protective immune response for many viruses such as Dengue virus^10,11^ or the previously mentioned BTV^12^ and African Horse Sickness Virus (AHSV) can also be loaded in the MS (Marín-López *et al*., manuscript in preparation).

We wanted to test our methodology against some of the main bottlenecks in protein expression for immune or other purposes: expression of membrane proteins, including glycoproteins, and expression of toxic/difficult proteins.

Glycoproteins are synthesized inside the endoplasmic reticulum (ER), where they acquire a modification by a core sugar moiety that is subsequently modified through the secretory route. Although the final sugar composition and structure is usually important for the function of eukaryotic proteins, the presence of complex sugars on the outer shell of enveloped viruses it is also used to evade the host immune system^13,14^. However, the initial addition of the core sugar moiety and the particular environment of the ER influences protein folding in many occasions^15^. On the other hand, many studies are impaired by the unavailability of methods to express proteins with inherent cell toxicity, as is the case for many membrane glycoproteins. As the IC-tagging methodology relies on the sequestration of the IC-tagged protein in MS inside the cell, it might be useful for expressing difficult proteins, as far as their toxicity is dependent on their cellular location.

Here we describe the successful adaptation of the IC-Tagging system to the ER, where we were able to load MS with the glycoprotein of vesicular stomatitis virus (VSV-G) and Rift Valley Fever Virus Gn ectodomain, both properly glycosylated. Enveloped viruses represent a challenge for subunit vaccine design, as they acquire a portion of the plasma membrane fully loaded with viral glycoproteins when they exit the cell. As the MS generated by IC-Tagging can be used as adjuvant-free subunit vaccines, the new ER-variant opens the possibility of producing safe and stable subunit vaccines against enveloped viruses. Additionally, we describe the adaptation of the IC-Tagging method to bacteria, where we were able to express the Islet-specific glucose-6-phosphatase catalytic subunit-related protein (IGRP), an antigen of enormous pharmaceutical interest as it is specifically targeted during the autoimmune response taking place in both the Non-Obese Diabetic (NOD) mice and type 1 diabetes (T1D) patients leading to the destruction of insulin-producing beta cells^16–19^. However, all the studies performed with IGRP were carried out using synthetic peptides, as IGRP expression remained elusive, because its cell toxicity when over-expressed^20^.

## Results

In order to produce muNS-Mi microspheres through the secretory route (Fig. 1b), we constructed a recombinant plasmid to direct the expression of the fusion protein sec-muNS-Mi, that bears the sequence of a typical secretory signal peptide fused to the N-terminus of the ARV-derived protein muNS-Mi (see methods). After transfecting DF-1 cells with the construct, we detected the MS by indirect immunofluorescence using muNS-specific antibodies on paraformaldehyde-fixed cells. As a control, DF-1 cells were transfected with a plasmid that expresses the previously characterized cytosolic version of muNS-Mi^5^. We could observe that sec-muNS-Mi produced intracellular microspheres that were smaller than the ones produced by its cytosolic counterpart (Fig. 1c, compare pictures 1 and 2). Such size difference might well be indicating their inclusion inside the ER as expected. However, the efficiency of the ER microsphere formation was lower than expected if compared with the efficiency in MS formation previously shown by muNS-Mi in the cytosol^5^. The secretory version produced only microspheres with a regular size and shape in approximately 25% of the transfected cells, while the other 75% showed either irregularly-shaped aggregates (Fig. 1c, picture 3), or not obvious microspheres (Fig. 1c, picture 4).

The lumen of the ER is a singular location with completely different conditions from the cytosol, and maybe an ER-specific activity or physicochemical characteristic was negatively influencing the formation of the MS. Thus, we investigated if any of the two main protein modifications that occur inside the ER, disulphide bond formation and glycosylation, were altering the structure of sec-muNS-Mi. We first checked disulphide bond formation by comparing cytosolic and sec-muNS-Mi by SDS-PAGE analysis in non-reducing conditions. We did not find any changes in the electrophoretic mobility of sec-muNS-Mi when comparing reducing and non-reducing conditions, what rules out the possibility of disulphide bond formation on muNS-Mi (Figure S1c, supplementary material). However, we did find a decreased electrophoretic mobility under non-reducing conditions of a GFP-fused muNS-Mi to which we added the same signal sequence used for muNS-Mi (sec-GFP-muNS-Mi, Figure S1b, supplementary material). This result suggests that sec-GFP-muNS-Mi contains disulphide bonds, what in turn suggests that the signal sequence attached to this protein, and also to muNS-Mi, is able to translocate its attached proteins to the interior of the ER. In spite of the obvious absence of disulphide bonds in sec-muNS-Mi, its apparent molecular size was bigger than its cytosolic counterpart, and the observed difference was too large to be explained by the only presence of uncut signal peptide (Fig. 1d, compare 1 and 2). On the other hand, the electrophoretic mobility of sec-muNS-Mi increased after digestion with N-glycosidase (Fig. 1d, compare 1 and 3), while no apparent difference could be observed in muNS-Mi after the same treatment (Fig. 1d, compare 2 and 4). Those results clearly indicated that sec-muNS-Mi is glycosylated and consequently, that the polypeptide is efficiently translocated into the ER.

**Figure 2:**
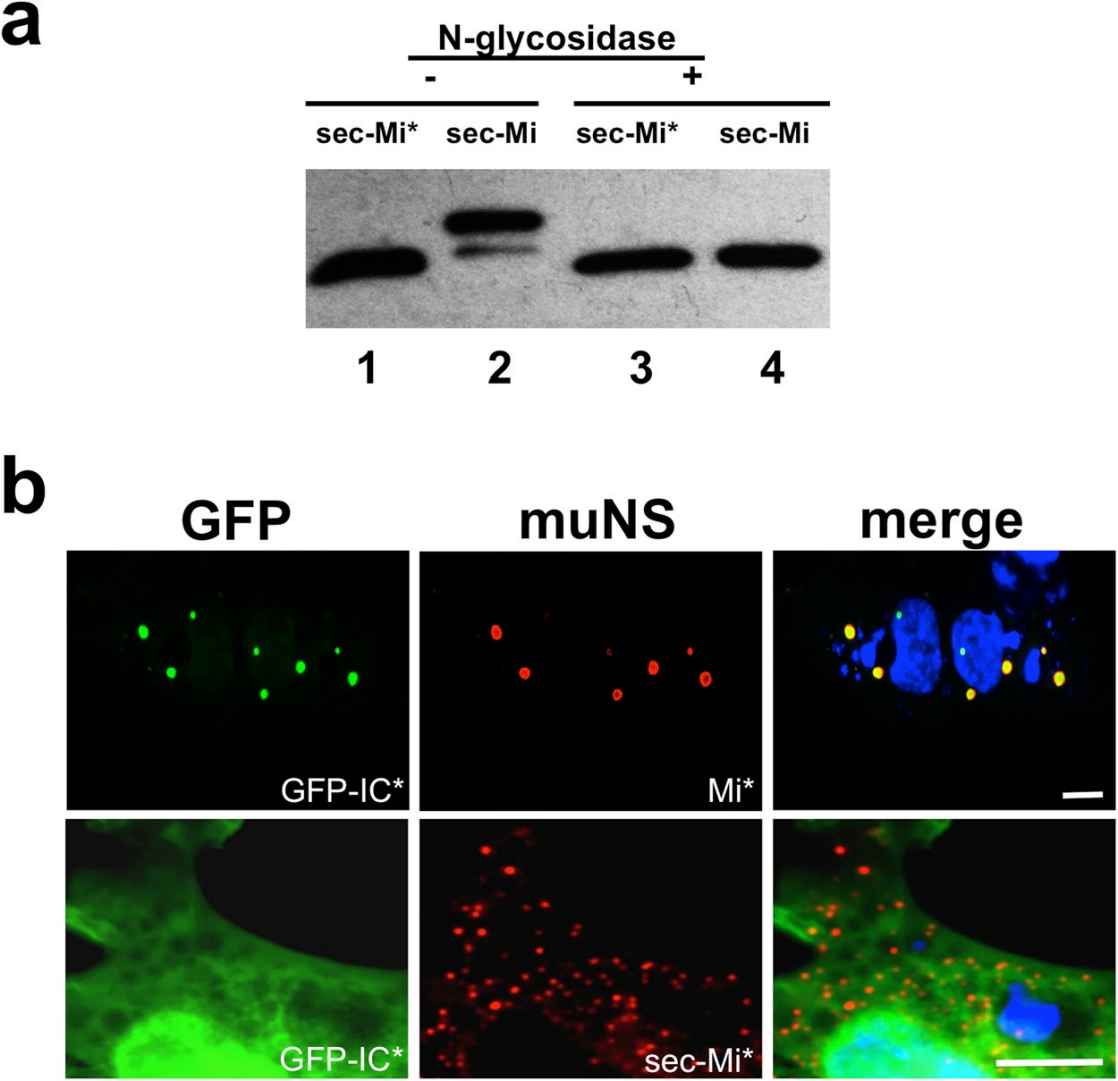
Characterization of mutated sec-muNS-Mi*. (**a**) Western-blot analysis of extracts from DF-1 cells transfected with expression plasmids for sec-muNS-Mi (sec-Mi, 2 and 4) or mutated sec-muNS-Mi (sec-Mi*, 1 and 3) either before (−) or after treatment with N-glycosidase (+). Full length Western-blot is shown in Figure S8. (**b**) Immunofluorescence analysis of DF-1 cells transfected with expression plasmids for muNS-Mi mutated on the IC domain (upper row, Mi*), or mutated sec-muNS-Mi (lower row, sec-Mi*), and both co-transfected with cytosolic mutated IC-tagged GFP (GFP-IC*). Specific anti-muNS antibodies were used to detect muNS-Mi* and sec-muNS-Mi* by indirect immunofluorescence (red). GFP fluorescence is seen in green, and nuclei were stained blue with DAPI. The white bar on the merged pictures corresponds to a distance of 5 µm.

When analysing the sequence of muNS-Mi, we detected a canonical N-glycosylation site (NVS) in residues 504–506 (referred to the sequence of the full-length muNS protein). This site falls just inside the IC-domain, crucial for the inclusion formation and the same used to target foreign proteins to muNS-Mi MS with the IC-Tagging methodology. We and others have previously reported that specific point mutations within the IC domain completely disrupt the ability of muNS to form intracellular inclusions^5,21^. Thus, it seems reasonable to think that the presence of voluminous sugar groups in that particular domain could hinder the formation of MS inside the ER. To test that hypothesis and try to overcome the low efficiency in forming MS, we decided to mutate the glycosylation site by changing the asparagine to a serine (NVS-SVS). We distinguish all the mutated constructs with an asterisk. As the IC domain is crucial for the formation of MS and also for the recruitment of foreign proteins to them, we decided to check first if the point mutation affected both activities by using the already characterized cytosolic versions of muNS-Mi and a IC-tagged protein^6^. The results are shown in Figure S2 of the supplementary material (see also Fig. 2b, upper row) and demonstrate that the mutation does not affect to any of the activities related to the IC domain. Furthermore, the recruitment of tagged proteins to either the wild-type or mutated muNS-Mi was equally effective when carrying a wild-type or a mutated IC-Tag.

To check if the mutation was effective in eliminating the glycosylation on sec-muNS-Mi*, we first expressed it transiently by transfection of DF-1 cells, and compared the electrophoretic mobility of the resulting cell extracts with those treated with N-glycosidase. The Western-blot analysis in Fig. 2a shows that sec-muNS-Mi* does not experiment any change in electrophoretic pattern after N-glycosidase treatment (compare lanes 1 and 3), while an evident increment of electrophoretic mobility occurs when the treatment is applied to the wild-type protein (compare lanes 2 and 4), indicating that the mutation was effective in eliminating the intra-ER glycosylation of sec-muNS-Mi*. The results shown in Fig. 2a also unequivocally demonstrate that the NVS site of the IC domain was the one and only working glycosylation site present in muNS-Mi.

We then looked at immunostained cells to check the appearance and efficiency of the ER-targeted MS formation driven by the mutated protein. In the lower row of Fig. 2b, it can be seen that the MS produced by sec-muNS-Mi* were smaller (diameter under 1 µm) than the ones generated by sec-muNS-Mi (Fig. 1c, picture 2, diameter usually over 1 µm), and also smaller than the cytosolic MS (Fig. 2b, upper row, diameter between 1 to 4 µm)). The general aspect of the ER-MS is very regular and the proportion of productively transfected cells carrying ER-MS is higher than 95%, similar to the efficiency of cytosolic MS formation by muNS-Mi, suggesting that IC-glycosylation was hindering the formation of the ER-MS driven by non-mutated sec-muNS-Mi.

Although the presence of glycosylations in sec-muNS-Mi clearly demonstrates its presence inside the secretory route, we performed an additional experiment to show it. Thus, we decided to co-express either the cytosolic or the ER-targeted muNS-Mi with a cytosolic IC*-tagged protein to check if the tagged polypeptide gets recruited to the respective MS. Figure 2b shows that cytosolic GFP-IC* gets efficiently recruited to muNS-Mi MS (upper row, compare GFP-IC* with Mi*) as expected. However, the small MS formed by sec-muNS-Mi* do not interact at all with GFP-IC* (Fig. 2b, lower row, compare GFP-IC* with sec-Mi*), again suggesting that they are in different compartments.

**Figure 3:**
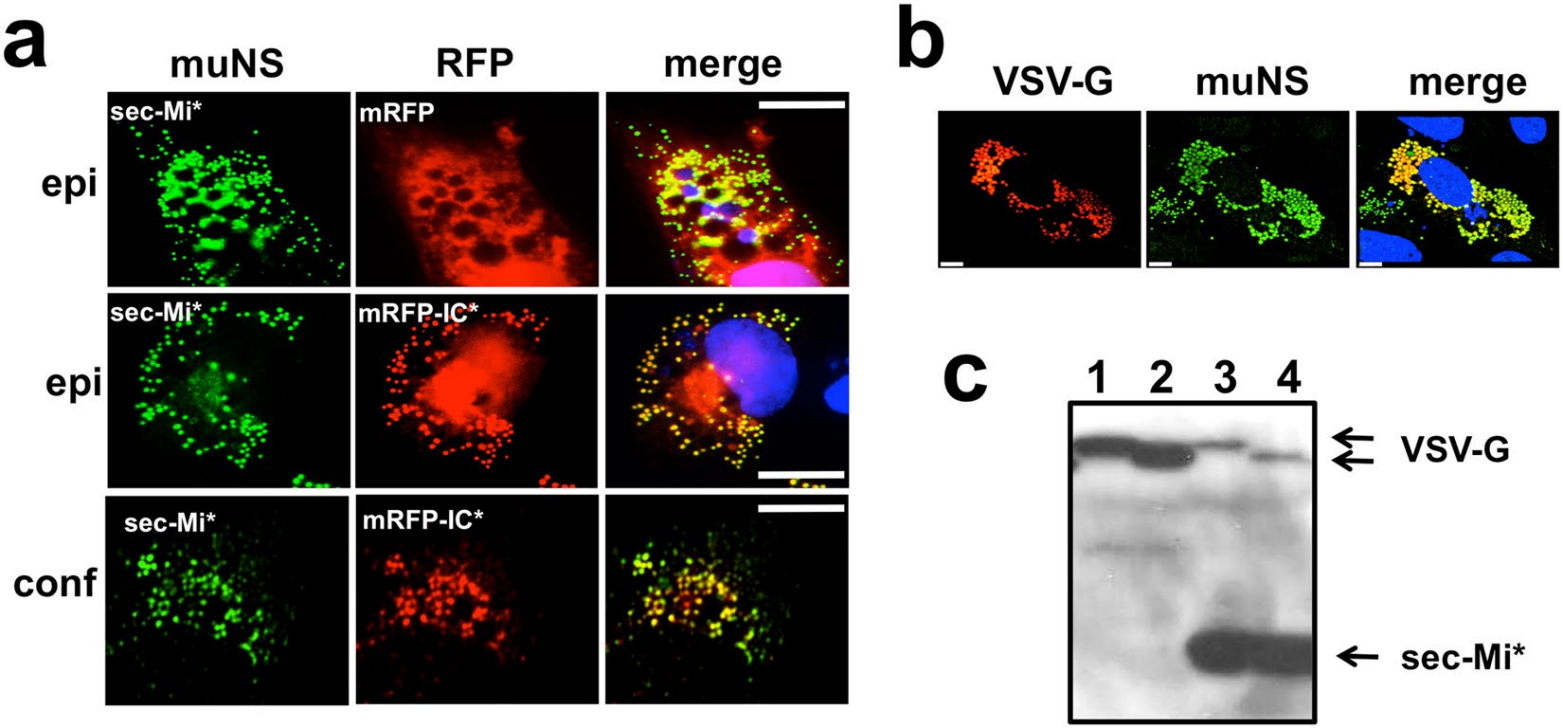
Characterization of protein re-localization to sec-muNS-Mi* MS inside the ER promoted by IC*-tagging. (**a**) Immunofluorescence analysis of DF-1 cells transfected with sec-muNS-Mi* (sec-Mi* in the figure) and co-transfected either with sec-mRFP (upper row) or sec-mRFP-IC* (middle and lower row). mRFP fluorescence is seen in red, while sec-muNS-Mi* is shown in green after detection with muNS-specific antibodies. Nuclei were stained blue with DAPI and shown in the merged images. The white bar represents a distance of 5 µm. The pictures of the upper and middle row were taken with an epi-fluorescence microscope (epi on the left of the figure). The lower row shows pictures of a slice acquired with a confocal microscope. (**b**) Confocal microscopy analysis of DF-1 cells transfected with sec-muNS-Mi* and co-transfected with a chimera formed by the ectodomain of the glycoprotein of VSV fused to mRFP and the IC-Tag (VSV-G). The presence of sec-muNS-Mi* was revealed by using muNS-specific antibodies (green). The VSV chimera was detected by the mRFP fluorescence. Nuclei were stained blue with DAPI and shown in the merged image. The white bar represents a distance of 5 µm. (**c**) Western-blot analysis performed with a mix of antibodies against the SV5 epitope and muNS. The samples came from extracts from DF-1 cells transfected with expression plasmids for a VSV chimera similar to the one described in b, but where the mRFP was replaced by the SV5 epitope (1 and 2) or co-transfected with sec-muNS-Mi* (3 and 4) either before (1 and 3) or after (2 and 4) treatment with N-glycosidase.

A remote possibility exists that, because of the presence of the secretory signal, the MS formed by sec-muNS-Mi* are still cytosolic, but assembled in such a way that are not able to capture IC-tagged proteins. To rule out that possibility and to further develop our methodology, we decided to construct an IC*-tagged reporter protein with a signal peptide at its N-terminus to direct its translocation into the ER and check if it can be loaded in the ER-MS. To avoid the use of antibodies, we decided to use the monomeric red fluorescent protein (mRFP) bearing a signal peptide at its N-terminus either untagged (sec-mRFP), or tagged in its C-terminus with IC* (sec-mRFP-IC*). We co-expressed both constructs independently with sec-muNS-Mi*, and analysed their expression and intracellular localization by immunofluorescence. The results shown in Fig. 3a unequivocally showed that, while there is no specific interaction between sec-muNS-Mi* and sec-mRFP (Fig. 3a, upper row, compare sec-Mi* with sec-mRFP), the presence of the IC* tag directs the recruitment of sec-mRFP-IC* to ER-MS generated by sec-muNS-Mi* (Fig. 3a, middle and lower rows, compare sec-Mi* with sec-mRFP-IC*). Co-localization between sec-Mi* and sec-mRFP-IC* showed typical Pearson R values above 0.9. We did not observe any apparent toxic effect produced by the expression of both proteins and the presence of the MS inside the ER, as is exemplified by the video included as supporting material (Movie 1). The time-lapse video shows a cell bearing ER MS loaded with sec-mRFP-IC*, recorded during 11 consecutive hours at 24 hours post-transfection and looking as healthy as the other non-transfected cells present in the same plate/field.

One of the main goals of developing a functional IC-Tagging system in the ER, is the possibility of loading glycosylated proteins in the ER-MS (Fig. 1b). In particular, as our MS were shown to work very well as subunit vaccines, it would be extremely appealing to simulate the particles of enveloped viruses by replacing the transmembrane domain of a viral glycoprotein by the IC*-Tag and generate MS fully loaded with the viral glycoprotein ectodomains. To test this idea, we focused on the glycoprotein of the rhabdovirus vesicular stomatitis virus (VSV-G), that mediates viral attachment and membrane fusion^22^. As a second example we chose the Rift Valley Fever Virus (RVFV) Gn Glycoprotein: one of the two glycoproteins present in the viral lipid envelope that is also used to contact the cell receptor^23^. To make them compatible with our ER-MS system, we made constructs where the respective transmembrane domains were completely removed and replaced with the IC*-tag. After co-expressing the chimeric proteins with sec-muNS-Mi* in DF-1 cells, we analysed their intracellular localization by confocal microscopy. The analysis showed that the chimeric IC*-tagged proteins completely co-localized with the sec-MS* (Figs 3b and S3 of the supplementary information), while no co-localization was evident in the absence of the IC* tag (Figure S3, supplementary information).

As the wild-type IC domain of protein muNS gets glycosylated in the ER, it could be assumed that IC*-tagged proteins loaded on them would also get glycosylated accordingly. To demonstrate that this is the case, we compared the glycosylation status of the VSV-G and Gn ectodomains, either when they were expressed alone or when loaded into ER-MS*. To assure that most of the expressed viral proteins would be incorporated in the MSs, we used a big excess of sec-muNS-Mi*. Notice that muNS-Mi and either VSV-G or Gn expression does not seem to interfere with each other as is shown in the Figure S4 of the supplementary material. The Western-blot analysis of untreated or N-glycosidase-treated extracts from transfected DF-1 cells revealed that the size of the untreated samples was exactly the same in both cases (Fig. 3c, compare lanes 1 and 3), as was the size-reduction after the treatment (Fig. 3c, compare lanes 2 and 4) showing that, as expected, the glycosylation patterns were unchanged by the inclusion of the VSV-G ectodomain in the ER-MS*. The same was also true for Gn ectodomain (Figure S5 supplementary information).

**Figure 4:**
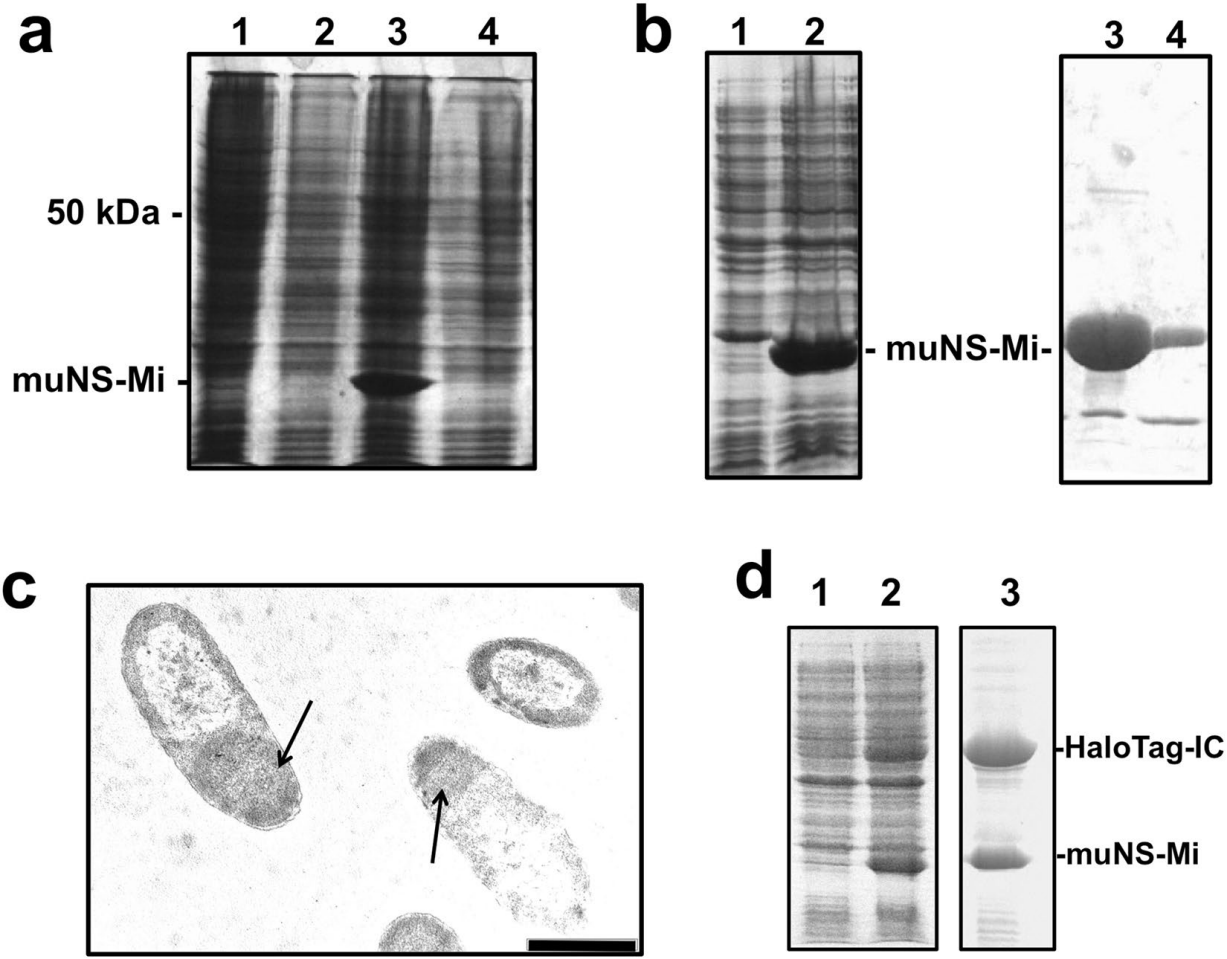
IGRP toxicity and development of IC-Tagging in bacteria. (**a**) Coomassie-blue PAGE analysis of extracts from Sf9 cells uninfected (1) or infected with or recombinant baculoviruses directing the expression of IGRP (2), muNS-Mi (3), or co-infected with the muNS-Mi and IC-IGRP baculoviruses (4). The position corresponding approximately to the size of muNS-Mi (21 kDa) is indicated at the left of the picture, as is the 50 kDa marker as a reference for the IC-IGRP approximate size (55 kDa). (**b**) Extracts from un-induced (1) or IPTG-induced bacteria (2) transformed with an expression plasmid containing the muNS-Mi gene were analyzed by PAGE and stained with Coomassie blue. The right side of the figure shows the supernatant (3) and pellet (4) of protein muNS-Mi purified from a sample similar to the one shown in lane 2 by EDTA treatment (see text). (**c**) The EM picture shows MS inside bacteria where the expression of muNS-Mi was induced. The arrows indicate the positions of the MS. The black bar represents a distance of 1 µm. (**d**) IC-tagging working in bacteria. Extracts from un-induced (1) and IPTG-induced (2) bacteria transformed with a dual recombinant plasmid that directs the simultaneous expression of proteins muNS-Mi and HaloTag-IC were analysed by PAGE and Coomassie-blue staining. Lane 3 shows a sample of the partial purification of MS from a sample similar to the one shown in lane 2. The positions corresponding to the calculated size of both proteins are indicated.

**Figure 5:**
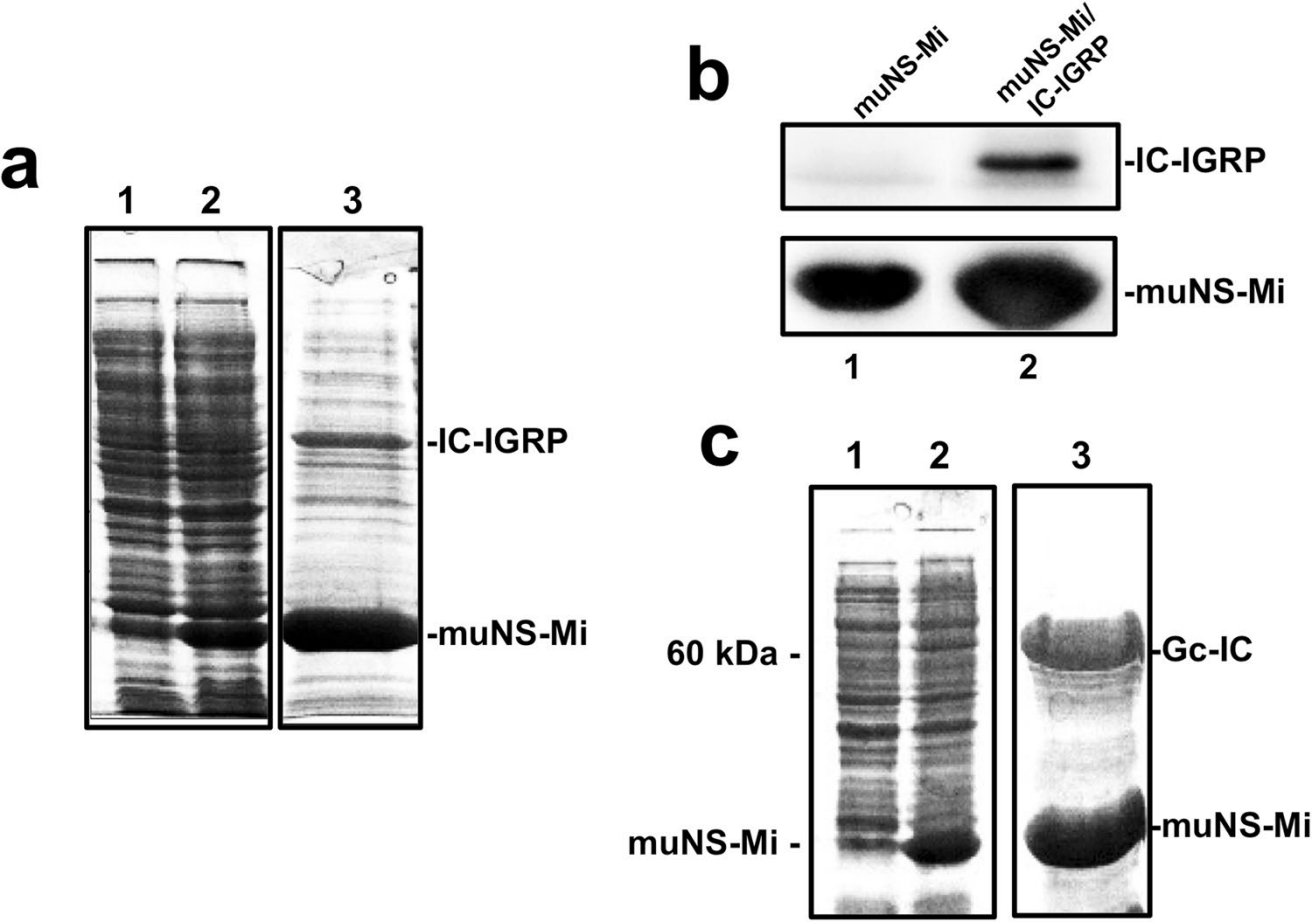
Expression of difficult-to-express proteins. (**a**) Extracts from un-induced (1) and IPTG-induced (2) bacteria transformed with a dual recombinant plasmid that directs the simultaneous expression of proteins muNS-Mi and IC-IGRP were analysed by PAGE and Coomassie-blue staining. Lane 3 shows the result of the concentration and partial purification of the MS present in a sample similar to the one shown in 2. The positions of the calculated molecular weights for both proteins are indicated. (**b**) Purified MS from bacteria expressing muNS-Mi (1, muNS-Mi), or simultaneously expressing muNS-Mi and IC-IGRP (2, muNS-Mi/IC-IGRP) were resolved on a polyacrylamide gel and subjected to Western-blot analysis using specific anti-muNS antibodies. The regions corresponding to the calculated molecular weights of both proteins are shown. Full length Western-blot is shown in Figure S9. (**c**) A similar analysis as shown in (**a**) was performed for the couple muNS-Mi and the ectodomain of the RVFV protein Gc tagged with IC.

For the next step, we decided to try the expression of a challenging membrane protein. We chose the diabetogenic auto-antigen IGRP because, in spite of its pharmaceutical relevance as being directly associated with the development of type 1 diabetes, to the best of our knowledge there is no example in the literature showing a significant level of expression of this protein. As our method confines the IC-tagged protein inside MS, it might help to relieve a possible toxic effect induced by the over-expression of this protein. We first tried to express the native, unmodified protein by conventional expression methods in bacteria and with the baculovirus expression system. As expected, we could not observe any band in Coomassie-stained PAGE gels that might suggest the expression of IGRP with neither of the two methods. Furthermore, IPTG-induction of bacteria transformed with the expression plasmid for IGRP produced a reduction in the optical density of the cultures, indicating that IGRP expression is toxic for *E. coli*. (not shown). In the baculovirus system is impossible to distinguish if the absence of expression is directly due to a toxic effect of the protein or to a previous selection for not expressing viruses during the generation of the recombinant baculoviruses. To try to solve this problem, we constructed a recombinant baculovirus for promoting the expression of IGRP with the IC-tag fused to its N-terminus, and we used it to infect Sf9 cells, or to co-infect the same cells with the recombinant baculovirus expressing muNS-Mi. Unfortunately, we were not only unable to detect the expression of IC-IGRP when expressed alone (Fig. 4a, lane 2), but also when co-expressed with muNS-Mi (Fig. 4a, lane 4). Furthermore, and to our surprise, the co-infection with the IC-IGRP baculovirus completely abolished the expression of muNS-Mi, thus completely eliminating the possibility of sequestering IGRP inside the MS (Fig. 4a, compare lanes 3 and 4), what suggests that in the baculovirus system, IGRP either is also toxic or at least has a negative effect on protein expression or the baculovirus replicative cycle. We decided then to adapt the IC-tagging method to bacteria, a completely different cell type. As our methodology requires the simultaneous expression of two different proteins, we chose the dual expression plasmid pDuet-1 (Novagen) that has two different polylinkers, both driven by the T7 promoter. We first introduced the sequence of muNS-Mi in the first polylinker, to create the plasmid pDuet-Mi. After induction with IPTG, we observed the expression of a 21 kDa protein on a Coomassie-stained gel (Fig. 4b, lane 2), and confirmed that it corresponds to muNS-Mi by Western-blot analysis (not shown). Electron microscope analysis of the IPTG-induced bacteria showed the presence of spherical inclusions with similar appearance to those found inside baculovirus-infected Sf9 cells, but smaller, with a diameter around 0.4 μm (Fig. 4c). Taking those results into account, we aimed first to identify if the spherical structures observed were typical bacterial inclusion bodies, similar to those often observed when over-expressing foreign proteins with expression plasmids, or if they are in fact well-ordered, muNS-Mi spheres. We have observed that the smallest spheres formed by muNS-Mi in the baculovirus system dismantle easily when deprived of any divalent cations (our unpublished results). The same is not true for bacterial inclusion bodies that usually need harsh conditions as high concentrations of urea or guanidinium chloride to get solubilized, although in some occasions, protein-specific methods involving mild conditions were also reported^24^. We then developed a simple purification method for the bacterial spheres based on the protocol previously developed for the MS produced with baculovirus (see methods). The purified MS were then incubated in a buffer lacking divalent cations, but including 0.5 mM EDTA. It is clearly seen that after 4 hours of incubation most of the protein was already solubilized (Fig. 4b, lanes 3 and 4). Increasing volumes and time produce the complete solubilisation without the need of detergents or denaturing agents, demonstrating that the nature of the aggregate is typical of the ordered muNS-Mi MS. We next tested if the IC-tag also directs the re-location of the tagged protein to the MS in prokaryotic cells. For that, we decided to introduce the sequence of the HaloTag domain, IC-tagged on its C-terminus, in the second polylinker of the plasmid pDuet-Mi. The IPTG induction of the bacteria transformed with the double-recombinant plasmid showed the expression of two proteins with the expected molecular weights of HaloTag-IC and muNS-Mi (Fig. 4d, lane 2). After MS purification as above, not only muNS-Mi, but also HaloTag-IC was purified (Fig. 4d, lane 3), showing that IC-tagged proteins associate with muNS-derived MS also in bacteria. Furthermore, the HaloTag was still active and able to react with coumarin-labelled HaloTag ligand, while no labelling at all occurred on the MS in the absence of HaloTag (Figure S6, supplementary material). Next, we proceeded to clone the sequence of the IC-tagged IGRP in the second polylinker of pDuet-Mi and check the expression after induction as for HaloTag-IC. Initially we were not able to clearly detect the expression of IC-IGRP in the total extracts of the induced bacteria (Fig. 5a, compare lanes 2 and 1). However, the presence of muNS-Mi was evident as a clear 21 kDa band, what prompted us to purify and concentrate the MS to check for the presence of IC-IGRP. As can be seen on lane 3 of Fig. 5a, a band of approximately 55 kDa, corresponding to the expected molecular weight of IC-IGRP, was evident on the Coomassie-stained gel. The identity of such band as IC-IGRP was confirmed by Western-blot (Fig. 5b, lane 2) and mass spectroscopy analysis (not shown). To confirm the bacterial method as able to hold the expression of reluctant proteins, we decided to test the system with a second problematic protein: the ecto-domain of the Gc glycoprotein of RVFV. We were unable to get any expression of this protein outside the ER, either in any bacterial expression system, recombinant baculovirus, or the baculovirus version of the IC-Tagging method. As before, we introduced the Gc-IC sequence in the second polylinker of plasmid pDuet-Mi. As can be seen in the Fig. 5, although we could not detect a clear band in total extracts of induced cells (Fig. 5c, lane 2), a big stained band with a molecular weight corresponding to Gc-IC was visible in the purified, concentrated MS. The protein co-purified with the MS has the expected molecular weight of Gc-IC and its identity was confirmed by Western-blot analysis (not shown).

## Discussion

The IC-Tagging method produces very stable, easy to purify, protein MS^25^. The method can be potentially used for many different applications, ranging from production of immobilized enzymes for industrial use, concentration of the components of multi-protein complexes, etc^6–8^. We have also recently shown that they also function as particulate, adjuvant-free subunit vaccines^9^. Subunit vaccines are safe vaccines lacking any genetic material, and usually composed by one or several proteins present at the surface of the pathogen. Although safer than inactivated or attenuated viruses, they usually have to be administered with immuno-stimulant agents called adjuvants, whose use is controversial due to its intrinsic toxicity. Some of the advantages of the MS as vaccines over other known methods, as for example VLPs, is their lack of architectural constraints, their simple purification protocol, their high stability and their ability to load non-structural viral proteins, those that are not components of the viral capsid. Viral non-structural proteins are usually more conserved than the structural ones, and when targeted by the immune system, often produce a broader, multi-serotype protection range. As an example, we have shown in a recent study that vaccination against only one AHSV non-structural protein confers multi-serotype protection (Marín-López *et al*. manuscript in preparation), while the protection reached by VLP vaccination is serotype-specific^26^. The amplitude of the response in such study is similar to the serotype-specific response generated by vaccination against the surface protein VP2, responsible of the viral contact with the cellular receptor. In the present study we have loaded MS with glycoproteins inside the ER, which can be used for several purposes. Taking into account our previous results, a particularly appealing application of the ER MS is the development of safer vaccines against enveloped viruses. As mentioned before, the development of subunit vaccines against enveloped viruses is challenging because of the presence on their surface of viral glycoproteins embedded in the viral envelope. Thus, envelope glycoproteins must be co-expressed with the structural proteins to produce enveloped VLPs for these viruses^27^. As an alternative, in particular for influenza virus, the production of empty “virosomes” has been reported. These are membrane vesicles obtained by the separation of the viral envelope from authentic purified viruses, eliminating the viral protein capsid and genome^28^. However, neither VLPs nor virosomes are a practical solution for industrial production of vaccines against these viruses, as they are complex to generate, not easy to purify and the presence of a lipid membrane makes them unstable under storage. Here we show the formation of MS inside the ER and the successful loading of glycoproteins on them, setting the basis for their use in the production of simple and effective subunit particulate vaccines against enveloped viruses, as well as other different applications that would need the use of glycoproteins. The initial expression of sec-muNS-Mi inside the ER produced mixed results, ranging from small and regular MS to unspecific aggregates. We found out that sec-muNS-Mi acquired a glycosyl moiety when expressed inside the ER, and that the glycosylated sequence was located within the IC domain that is part of muNS-Mi and constitutes the working tag of the IC-Tagging methodology. An asparagine to a serine (NVS-SVS) point mutation completely abolished such glycosylation, restoring the ability of sec-muNS-Mi to form inclusions inside the ER. Such mutation had no negative effect on the IC-Tagging methodology, as we showed that mutated and wild-type forms of muNS-Mi are both able to capture proteins tagged either with the wild-type or mutated IC-Tag with no difference in efficiency in the cytosol. Using a version of mRFP that gets translocated in the ER (sec-mRFP), we demonstrated that proteins can be specifically loaded in the ER-MS by tagging them with the IC-Tag. The wild-type form of muNS-Mi gets fully glycosylated inside the ER, what already shows that glycosylation and inclusion in ER-MS are compatible. However, we went a step forward by showing that we can load the IC-tagged ectodomains of VSV-G and RVFV Gn glycoproteins, and that they get fully glycosylated. Furthermore, the glycosylation level observed was independent on the presence or absence of an excess of muNS-Mi, indicating that the inclusion in the MS does not affect to the degree of glycosylation of the IC-Tagged protein, at least in the case of VSV-G.

IGRP is an important antigen in the development of T1D in humans, a disease where the immune system of the patients specifically destroys the insulin-producing cells of the pancreatic islets of Langerhans^19^. Thus, its production and purification is encouraged in order to carry out studies of the autoimmune response in diabetic patients or even its possible use as a tolerance-inducing vaccine that could prevent the development of T1D. The inability to produce appreciable amounts of IGRP has greatly limited such studies.

There is hardly any publication that mentions the expression and purification of this protein, and in no case the protein produced is shown in appreciable amount. Thus, in one of these publications^29^, the authors mention that they have cloned the open reading frame corresponding to murine IGRP and expressed the protein as a fusion with beta-galactosidase. Although the authors claimed to purify IGRP to obtain antibodies by immunization of rabbits, no proof of the expression and/or purification is shown. A large majority of the studies used transient expression in different cell lines either to determine the genomic elements that control the expression of the gene or to determine a hypothetical enzymatic activity of the protein but without making any attempt of purification and showing no level of expression at all.

It is interesting to note that most of these studies have been unable to detect an increase in phosphatase activity hypothetically due to IGRP, with the exception of one of them^30^ where the authors claim to have expressed and subsequently purified IGRP using the baculovirus system, indicating that the protein presented phosphatase activity. The authors show an analysis by Western blot using anti-histidine antibodies and no stained gel, to demonstrate the expression of IGRP, what indicates that the level of expression and purification using this system must be quite low. The same authors also indicate that their various attempts to purify the protein produced a loss of the observed enzymatic activity what, together with the fact that the assays were performed with extracts from baculovirus-infected insect cells that are usually full of phosphatase activity, question those results.

Our initial attempts to express IGRP by conventional bacterial or baculovirus systems failed, possibly due to the toxic effects of this protein when over-expressed. Furthermore, when we tried to express IGRP with our IC-Tagging method in the baculovirus expression system, not only could not detect expression of IGRP but the expression of the MS-forming protein muNS-Mi was absolutely abolished, reassuring its negative effect on protein expression. The protein sequestration of IC-tagged proteins might be a possible way to express toxic or reluctant to express proteins by their re-location to MS, but such re-location should be rapid and efficient to avoid as soon and as thorough as possible the possible noxious effect. Bacteria have a small and non-compartimentalized intracellular space and have also a completely different metabolic framework than eukaryotic cells. Also, the absence of ER and the use of different mechanisms to translocate their proteins to the membrane give an additional chance for a eukaryotic membrane protein to interact with muNS-Mi, while inside a eukaryotic cytosol might exist a competition between its recruitment to the MS and its engagement with the ER translocon. Thus, we thought of checking if those characteristics might make a difference in the expression of a difficult protein as IGRP. We first demonstrated that the IC-Tagging method works in bacteria: muNS-Mi forms MS and the IC-tagged proteins are recruited to them, allowing their easy co-purification. We also showed that the MS were not typical bacterial inclusion bodies because, exactly as the original baculovirus-produced MS, they easily dismantled when deprived of divalent cations. We do not know exactly why this happens, but is possible that the absence of divalent cations modifies the buffer conditions in a way that alters the electrostatic interactions between muNS-Mi monomers crucial for the formation of MS. The first thing that we observed when co-expressing IGRP and muNS-Mi in bacteria was that the expression of muNS-Mi was not affected, showing that the toxicity of IGRP was different in the prokaryote model. However, even in the presence of a good amount of muNS-Mi, initially we did not observe a good IGRP expression after IPTG induction. The presence of muNS-Mi, indicating that the MS were forming, prompted us to purify and concentrate them in an attempt to rescue any possible IC-tagged IGRP that might be attached to them. After MS concentration, a band corresponding to IGRP was evident on a Coomassie stained gel, whose identity was confirmed first by Western-blot analysis and then, unequivocally, by mass-spectrometry. To the best of our knowledge, this is the first time that an appreciable amount of IGRP is shown on a stained gel, opening the possibility of performing the above-mentioned studies on its implication on T1D development and a putative T1D-relieving vaccine.

Although we are aware that IC-Tagging might not work as a universal method to express toxic or difficult proteins, we wanted to test its capacity with at least a second example. In the course of the production of glycoproteins with the IC-Tagging methodology, we faced problems when trying to express the ectodomain of the RVFV Gc glycoprotein outside the ER to use it as a control for some experiments. The Gc ectodomain has been successfully expressed before, and even its structure was already solved^31^, but always expressing the glycoprotein inside the ER, keeping the protein in its natural location. We were not able to express it outside the ER with the baculovirus system, either in the absence or the presence of the MS, possibly reflecting a stabilizing effect of the intra-ER glycosylation or ER-chaperone-assisted folding^15^. It is possible that in the absence of a proper intra-ER folding, the protein gets aggregated and quickly discarded by the cell. As shown in the results section, again we were able to produce a big amount of the Gc ectodomain with the bacterial version of our methodology, what reinforces its capacity for the production of proteins whose expression is inherently difficult.

In summary, we developed two new versions of our IC-Tagging methodology inside eukaryotic cells ER and inside bacteria. With the new versions we are able to load glycoproteins and reluctant full membrane proteins in the protein MS produced by the ARV muNS-Mi protein, that can be used for multiple purposes, including adjuvant-free subunit vaccines.

## Materials and Methods

### Cells and antibodies

DF-1 cells^32^ were grown in monolayers in medium D-MEM supplemented with 5% foetal bovine serum (FBS), 2% of 4 mM L-glutamine and 1% antibiotic mix (penicillin and streptomycin). Sf9 cells (Thermo Fisher Scientific) were grown in suspension in medium SF-900II supplemented with 5% foetal bovine serum (FBS), 2% of 4 mM L-glutamine and 1% antibiotic mix (penicillin and streptomycin). *E. coli* strain XL1-Blue (Stratagene, La Jolla, California) was used to grow and purify plasmids. BL21-CodonPlus-RP (DE3) (Agilent Technologies) was used for protein expression.

Rabbit polyclonal antiserum against avian reovirus S1133 muNS protein was raised in our laboratory^4^. Monoclonal antibody specific for SV5 Tag was obtained from Life Technologies. The following secondary antibodies were used as appropriate for different experiments: Alexa Fluor 594 conjugated antibodies against mouse or rabbit IgG; Alexa Fluor 488 conjugated antibodies against rabbit IgG (Invitrogen, Barcelona, Spain). Peroxidase-conjugated goat anti-rabbit IgG antibodies (Sigma-Aldrich, Madrid, Spain) were used for Western-blot analysis.

### Transfections and IF microscopy

Transfections of cell monolayers were done with Lipofectamine 2000 (Invitrogen, Barcelona, Spain), according to the manufacturer´s instructions. Transfected cells were incubated at 37 °C for 24 h, unless otherwise stated.

For indirect immunofluorescence microscopy, cell monolayers grown on coverslips were transfected, and, at the indicated times, the monolayers were washed three times with PBS and fixed for 10 min with 4% paraformaldehyde in PBS. Paraformaldehyde-fixed cells were washed twice with PBS, incubated for 5 min in permeabilizing buffer (0.5% Triton X-100 in PBS), and then blocked in PBS containing 2% bovine serum albumin for 30 min at room temperature. Then, the cells were incubated for 1 h at room temperature with primary antibodies diluted in blocking buffer. After three washes with PBS, the cells were incubated for 30 min with secondary antibodies and DAPI. Coverslips were then washed and mounted with Mowiol. The widefield pictures were taken either with an Olympus DP-71 digital camera mounted on an Olympus BX51 fluorescence microscope or with a Zyla 4.2 camera (Andor) mounted on a Nikon TiE microscope. Living cells imaging and time-lapse recording were performed on a Nikon TiE microscope equipped with a Zyla 4.2 camera and an OKO-lab incubator. Confocal images were captured on an Andor Dragonfly spinning disk confocal system mounted on a Nikon TiE microscope equipped with a Zyla 4.2 PLUS camera (Andor). Images were processed either with Imaris (Oxford Instruments), NIS elements (Nikon), or Adobe Photoshop (Adobe System, California, USA).

### N-glycosidase treatment and immunoblotting

Cells were collected on a buffer containing 50 mM TrisHCl (pH 8.0), 150 mM NaCl, and 0.5% SDS. For N-glycosidase treatment, 10 μl of cell extract was added to a mix of 2 units of N-glycosidase F (Roche), 6 μl of the same buffer, 3 mM DTT and 2 μl of 10% NP-40. Samples were incubated at 37 °C for 1 h and then analysed by Western-blot with the corresponding antibodies.

For Western-blot analysis, cell extracts were resolved by SDS-PAGE and proteins in unfixed gels were transferred to PVDF membranes (Immobilon-P, Millipore, Madrid, Spain) for 1 h at 100 V in a semidry blotting apparatus (Bio-Rad, California, USA). Protein bands were detected by incubation with specific antibodies and HRP-conjugated secondary antibodies using the Immobilon Western Chemiluminiscent HRP substrate (Millipore, Madrid, Spain).

### Protein expression in bacteria and MS purification

BL21 CodonPlus-RP (DE3) bacteria transformed with the expression plasmid were diluted and incubated at 37 °C with shaking to reach at OD_600_~ 0.4–0.6. Expression was induced with 1 mM IPTG and incubated at 37 °C with shaking for 3 h. Induced cultures were centrifuged at 3200 g and the pellet washed twice in PBS, resuspended in 1/10 volume of lysis buffer (0.25% Tween-20, 1 mM DTT, 200 mM NaCl, 20 mM Tris pH 7.5, 2 mM MgCl_2_) and frozen. The thawed pellet was sonicated and centrifuged at 2700 g, 5 min at 4 °C, washed in RB + buffer (10 mM Hepes pH 7.9; 10 mM KCl; 5 mM MgCl_2_) containing 0.5% Triton X-100 once, washed in RB+ fivefold, and finally resuspended in a small volume of RB+.

### EM analysis of bacteria

IPTG-induced Bacteria were centrifuged at 2700 g, 5 min at 4 °C, washed 3 times in PBS and incubated with fixative solution (2% glutaraldehyde in PBS) for 20 min at room temperature. Bacteria were postfixed in 1% O_4_Os for 1 h and embedded in 2% agarose to form a compact pellet. Pellets were dehydrated in increasing concentrations of ethanol and included in EPON. Ultra thin sections were obtained with a Leica Ultracut UCT ultramicrotome. Grids are finally stained with 2% Uranyl Acetate and Reynold Lead Citrate and observed in a TEM JEOL JEM1011.

### Plasmid constructions

The following plasmids have been described previously: i) pCINeo-muNS^4^ ii) GFP-muNS (477–542)^5^ iii) pcDNA (D-BssH)-secil-BAP-HLA-SV5 with a signal peptide was generous gift from Dr. Oscar Burrone (ICGEB-Trieste, Italy) iv) pEGFP-C1-M3 (448–635)^5^v) pCMV-M4 34 was a^33^ generous gift fom Alejandro Brun (CISA-INIA, Valdeolmos, Madrid, Spain).

#### sec-muNS-Mi

The plasmid pCINeo-muNS was subjected to PCR amplification to obtain the sequence of muNS-Mi. The PCR product was cloned into the plasmid pcDNA (D-BssH)-secil-BAP-HLA-SV5 to generate pcDNA sec-muNS-Mi that expresses muNS-Mi fused to a signal peptide (sec).

#### sec-mRFP

The sequence of the monomeric Red Fluorescent Protein was PCR-amplified from plasmid ptfLC3^34^ (a gift from Tamotsu Yoshimori, Addgene plasmid # 21074). The PCR product was digested and cloned into the plasmid pcDNA (D-BssH)-secil-BAP-HLA-SV5 to generate the plasmid pcDNA sec-mRFP to express mRFP fused to a signal peptide.

#### sec-mRFP-IC

To express the mRFP protein fused to the IC domain inside the ER, the muNS IC domain was obtained by PCR from the recombinant plasmid pCINeo-muNS and cloned into the plasmid pcDNA sec-mRFP to generate pcDNA sec-mRFP-IC.

#### sec-GFP-muNS-Mi

The sequence of the EGFP-muNS-Mi fusion protein was obtained by PCR amplification from plasmid pEGFP-C1-M3 (448–635) and cloned into the plasmid pcDNA (D-BssH)-secil-BAP-HLA-SV5 to generate pCDNA sec-GFP-muNS-Mi that expresses GFP-muNS-Mi fused to a signal peptide.

#### sec-SV5

The SV5 sequence was obtained by PCR from plasmid pcDNA (D-BssH)-secil-BAP-HLA-SV5 and cloned into the plasmid pcDNA 3.1 Zeo+ to generate plasmid pcDNA sec-SV5.

#### sec-SV5-Gn

The ectodomain of Rift Valley Fever Virus Gn protein was obtained by PCR from plasmid pCMV-M4 cloned into the plasmid pcDNA sec-SV5 to generate pcDNA sec-SV5-Gn.

#### sec-SV5-Gn-IC

The IC domain was obtained by PCR from plasmid pCINeo-muNS and cloned into the plasmid pcDNA sec-SV5-Gn to generate pcDNA sec-SV5-Gn-IC.

#### sec-VSV-G-SV5

The ectodomain of the VSV glycoprotein (VSV-G) was obtained from plasmid pCMV-VSV-G^35^ (gift from Bob Weinberg, Addgene plasmid # 8454) by PCR amplification and cloned into the plasmid pcDNA 3.1 Zeo+ to generate pcDNA sec-VSVG. The SV5 sequence was fused to the sec-VSV-G with oligonucleotide adapters, that were digested and cloned into the plasmid pcDNA 3.1 Zeo+ sec-VSVG to generate sec-VSV-G-SV5.

#### sec-VSV-G-SV5-IC*

The IC domain was obtained by PCR from plasmid pCINeo-muNS* and cloned into the plasmid pcDNA 3.1 sec-VSV-G-SV5 to generate sec-VSV-G-SV5-IC*.

#### sec-VSV-G-mRFP

The sequence of the ectodomain of VSV glycoprotein (VSV-G) was obtained by PCR from plasmid pCMV-VSV-G. The PCR product was digested and cloned into the plasmid pcDNA 3.1 Zeo+ to generate pcDNA 3.1 sec-VSV-G. Then, mRFP sequence was obtained by PCR from plasmid ptfLC3 and cloned into the plasmid pcDNA 3.1 sec-VSV-G to generate pcDNA 3.1 sec-VSV-G-mRFP.

#### sec-VSV-G-mRFP-IC*

The sequence of the IC domain was obtained by PCR from plasmid pCINeo-muNS* and cloned into the plasmid pcDNA 3.1 sec-VSV-G-mRFP to generate sec-VSV-G-mRFP-IC*.

#### Bacterial muNS-Mi

To express muNS-Mi protein in bacteria, the sequence of muNS-Mi was obtained from the recombinant plasmid pCINeo-muNS by PCR amplification and cloned into the MCS 1 of the plasmid pET Duet-1 to generate pET Duet -Mi.

#### Dual-muNS-Mi/IC-IGRP

To express IC-IGRP in bacteria, we obtained the sequence of IGRP by PCR amplification using the PCR-Ready Human Pancreas cDNA (Ambion) as template. The amplified product was cloned into plasmid pFastBac, to obtain plasmid pFASTBac-IGRP. Then, the sequence of the muNS IC domain was amplified from plasmid pCINeo-muNS and cloned in frame in plasmid pFASTBac-IGRP, to obtain plasmid pFASTBac-IC-IGRP. The sequence of the fusion protein was again PCR-amplified and cloned into the MCS2 of the plasmid pET Duet 1. muNS-Mi to generate the pET Duet 1. muNS-Mi 2. IC-IGRP.

#### Dual-muNS-Mi/HaloTag-IC

The recombinant plasmid pFastBac HaloTag-IC was subjected to PCR amplification and the resulting product was digested and cloned into de MCS2 of the plasmid pET Duet 1.muNS-Mi to generate the pET Duet 1.muNS-Mi 2. HaloTag-IC.

#### muNS-Mi/GC-IC

To express the ectodomain of Rift Valley GC protein fused to the IC domain in bacteria, the recombinant plasmid pFastBac GC-IC was subjected to PCR amplification to obtain the sequence of the Gc ectodomain, that was cloned into the MCS2 of plasmid pET Duet 1. muNS-Mi to generate pET Duet 1.muNS-Mi 2.GC-IC.

### Mutagenesis

Single-aminoacid mutations were generated in the different plasmids indicated in the text using QuikChange Site-Directed Mutagenesis Kit (Stratagene).

No datasets were generated or analysed during the current study.

## Electronic supplementary material

Supplementary Information

Video 1
