# Supplementary material for: IC-Tagging methodology applied to the expression of viral glycoproteins and the difficult-to-express membrane-bound IGRP autoantigen
Source: Sci Rep. 2018 Nov 2;8:16286. doi: 10.1038/s41598-018-34488-3 (PMC6214907; doi:10.1038/s41598-018-34488-3)
Supplement: Supplementary file 1 — Supplementary Information [file 41598_2018_34488_MOESM1_ESM.pdf]

Supplementary Material for the manuscript:

IC-Tagging methodology applied to the expression of viral glycoproteins and the difficult-to-express membrane-bound IGRP autoantigen

Authors: Natalia Barreiro-Piñeiro[1], Irene Lostalé-Seijo[1,2], Rubén Varela-Calviño[3], Javier Benavente[1] and José M. Martínez-Costas[1]*

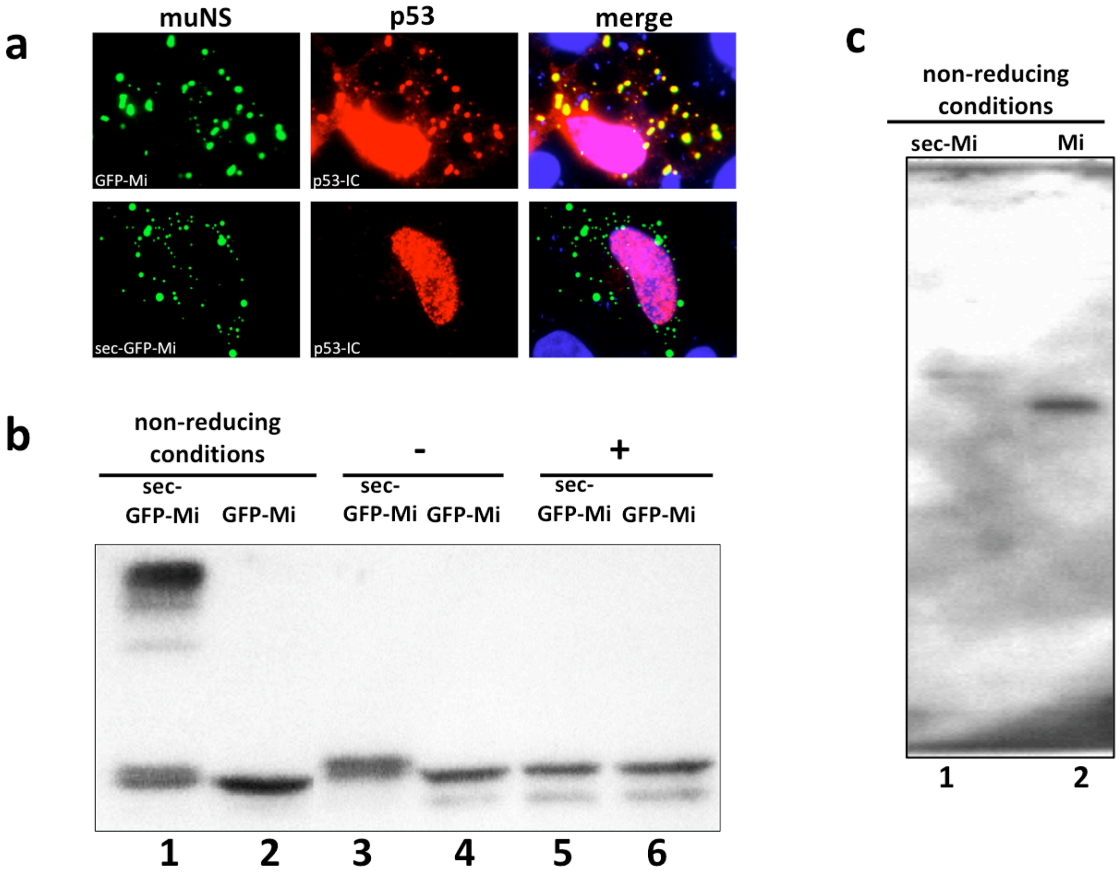

Figure S1: Expression of sec-GFP-muNS-Mi inside the ER. a. Comparison of the co-expression of IC-tagged p53 either with cytosolic (GFP-Mi, upper row) or intra-ER (sec-GFP-Mi, lower row) GFP-muNS-Mi. DF-1 cells transfected with plasmids directing the expression of the proteins indicated in the figure were fixed at 24 hours post-transfection and immunostained with specific anti-p53 antibodies (red). muNS is seen in green due to the green fluorescence of GFP, while nuclei were stained blue with DAPI. As observed, sec-GFP-muNS-Mi is able to make MS inside the ER, that do not capture cytosolic IC-tagged p53. **b**. **Analysis of the intra-ER post-translational modifications.** Extracts from DF-1 cells transfected with expression plasmids for GFP-muNS-Mi (GFP-Mi, lanes 2, 4 and 6) or sec-GFP-muNS-Mi (sec-GFP-Mi, lanes 1, 3 and 5) were subjected to PAGE eliminating the 2-mercaptoethanol from the PAGE sample buffer (non-reducing conditions), or either before (-) or after (+) treatment with N-glycosidase. The presence of GFP-muNS-Mi and sec-GFP-muNS-Mi were revealed by western-blot analysis using muNS-specific antibodies. As can be seen in the samples analyzed in non-reducing conditions, disulphide bond formation inside the ER generates higher MW bands of sec-GFP-muNS-Mi. Also, the glycosidase treatment of sec-GFP-muNS-Mi generates a faster-migrating band, indicating the intra-ER glycosylation of the protein. **c. Non-reducing conditions analysis of muNS-Mi and sec-muNS-Mi.** As sec-GFP-muNS-Mi was shown to contain disulphide bridges, a similar analysis was performed on muNS-Mi, by subjecting the samples to PAGE in the absence of 2-mercaptoethanol. As can be seen, the gel revealed no additional high molecular weight

band, demonstrating that no disulphide bridges are formed in the muNS-Mi moiety inside the ER.

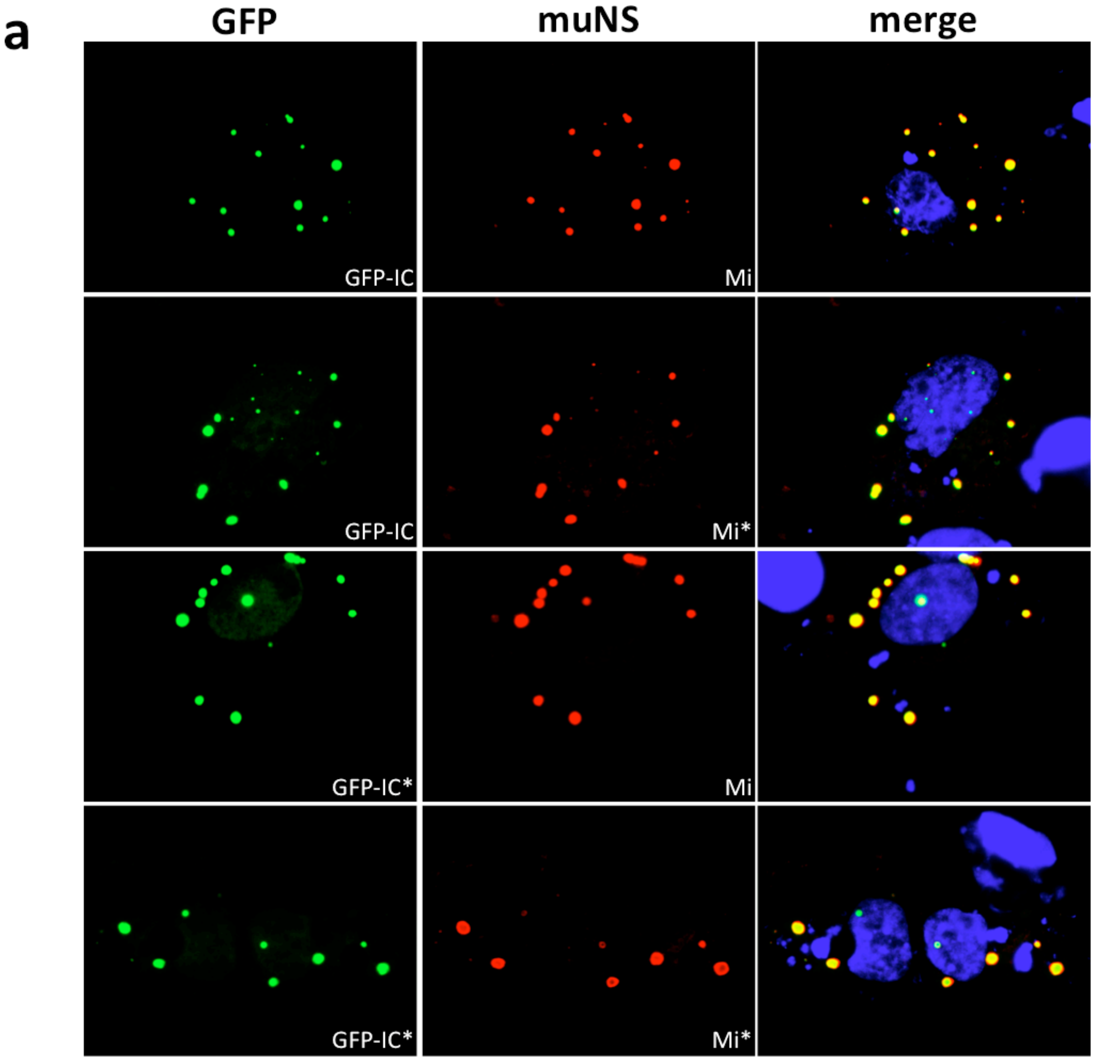

Figure S2: The NVS to SVS point mutation in the IC domain does not affect to MS formation or IC-tag capture. Inmunofluorescence analysis of DF-1 cells transfected with different combinations of mutated (GFP-IC* and muNS-Mi*) and non-mutated (GFP-IC and muNS-Mi). GFP-IC fluorescence is seen in green, while muNS-Mi (Mi in the figure) is shown in red after detection with muNS-specific antibodies. Nuclei were stained blue with DAPI and shown in the merged images. It is clearly seen that the IC-tagged proteins are equally recruited to the MS independently of the presence or absence of the NVS to SVS mutation (marked by an asterisk) either in the muNS-Mi protein or the IC-tag.

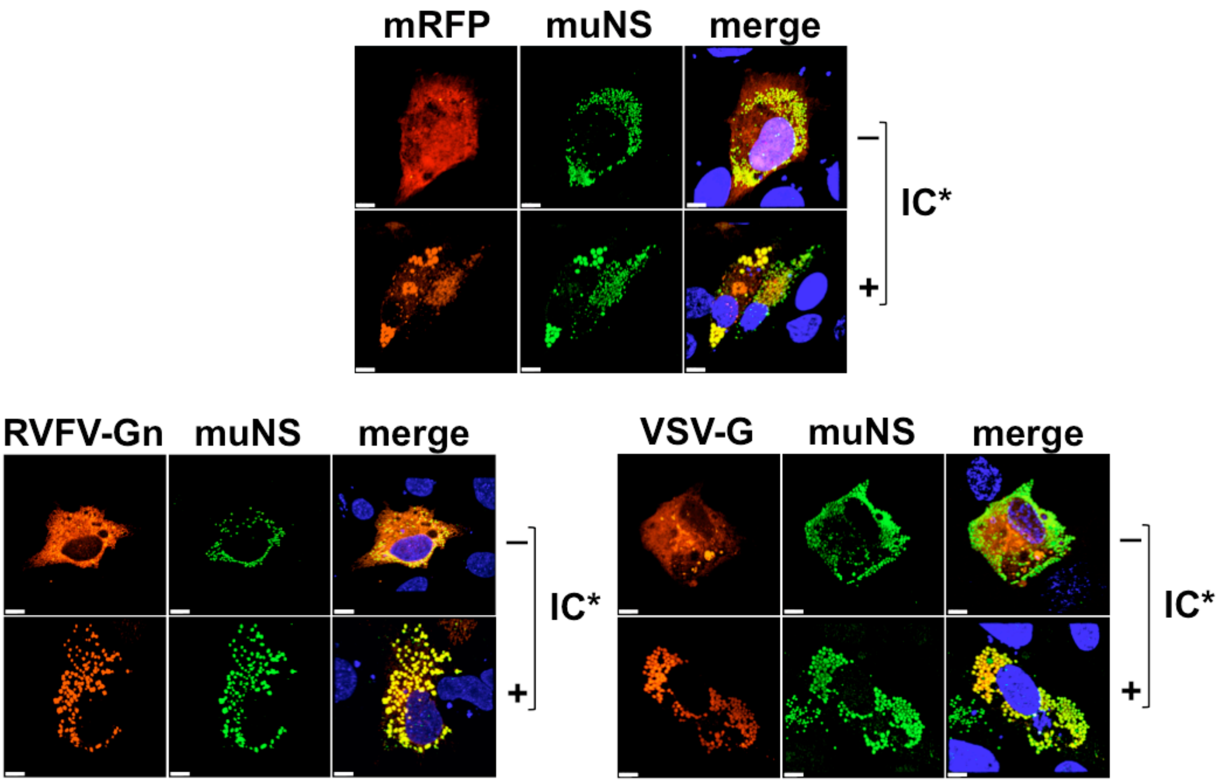

Figure S3: Integration of IC*-tagged proteins in MS inside the ER. The figure shows confocal microscopy pictures of DF-1 cells transfected with combinations of sec-muNS-Mi* and either a version of mRFP bearing a signal sequence at its N-terminus (upper panel); or the ectodomain of the RVFV Gn glycoprotein bearing a signal sequence at its N-terminus followed by the SV5 epitope (see sec-SV5-Gn on methods, RVFV-Gn on the picture of the lower left panel); or the ectodomain of the VSV-G glycoprotein bearing a signal sequence on its amino terminus followed by the mRFP moiety (see sec-mRFP-VSV-G on methods, VSV-G on the picture of the lower right panel). The three chimeras were expressed with (+) or without (-) the mutated IC-tag as indicated at the right of each panel. The presence of muNS was detected with specific antibodies against the viral protein, while mRFP fluorescence produced a red signal on the upper and lower right panels. Antibodies against SV5 epitope were used to detect RVFV-Gn protein. The white bar corresponds to a distance of 5 micrometers.

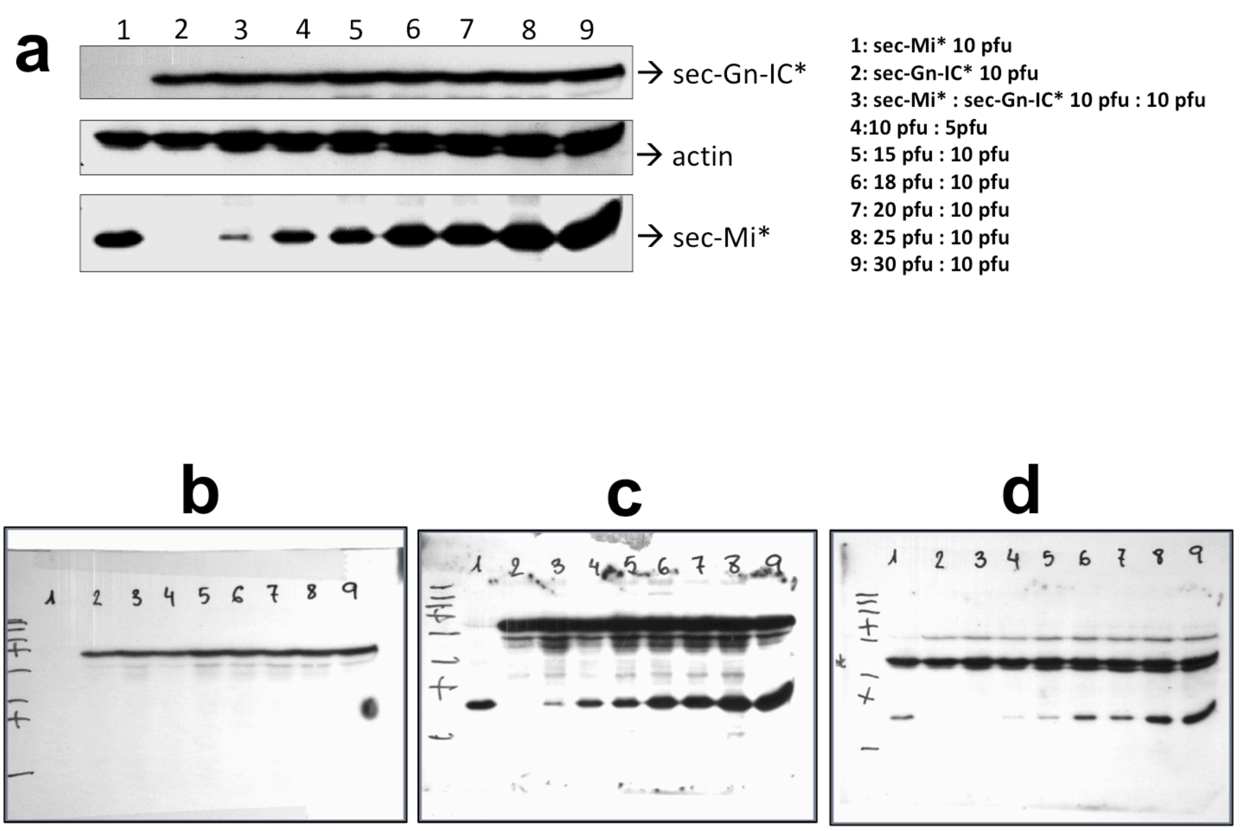

Figure S4: Co-habitation of sec-musNS-Mi* and sec-SV5-Gn inside the ER. a.Composite figure made with the full-length Western-blots shown in b, c and d- Western-blot analysis of DF-1 cells transfected (lines 1 and 2) and co-transfected (lines 3-9) with a fixed amount of a plasmid expressing sec-SV5-Gn-IC* (top panel) and increasing amounts of sec-muNS-Mi* (lower panel) (See also figure S3 and methods). Antibodies against muNS and SV5 were used to detect sec-muNS-Mi* (full length Western-blot in **c**) and sec-SV5-Gn-IC* (full length Western-blot in **b**) respectively. Actin was also detected as loading control (middle panel, full length Western-blot in **d**).

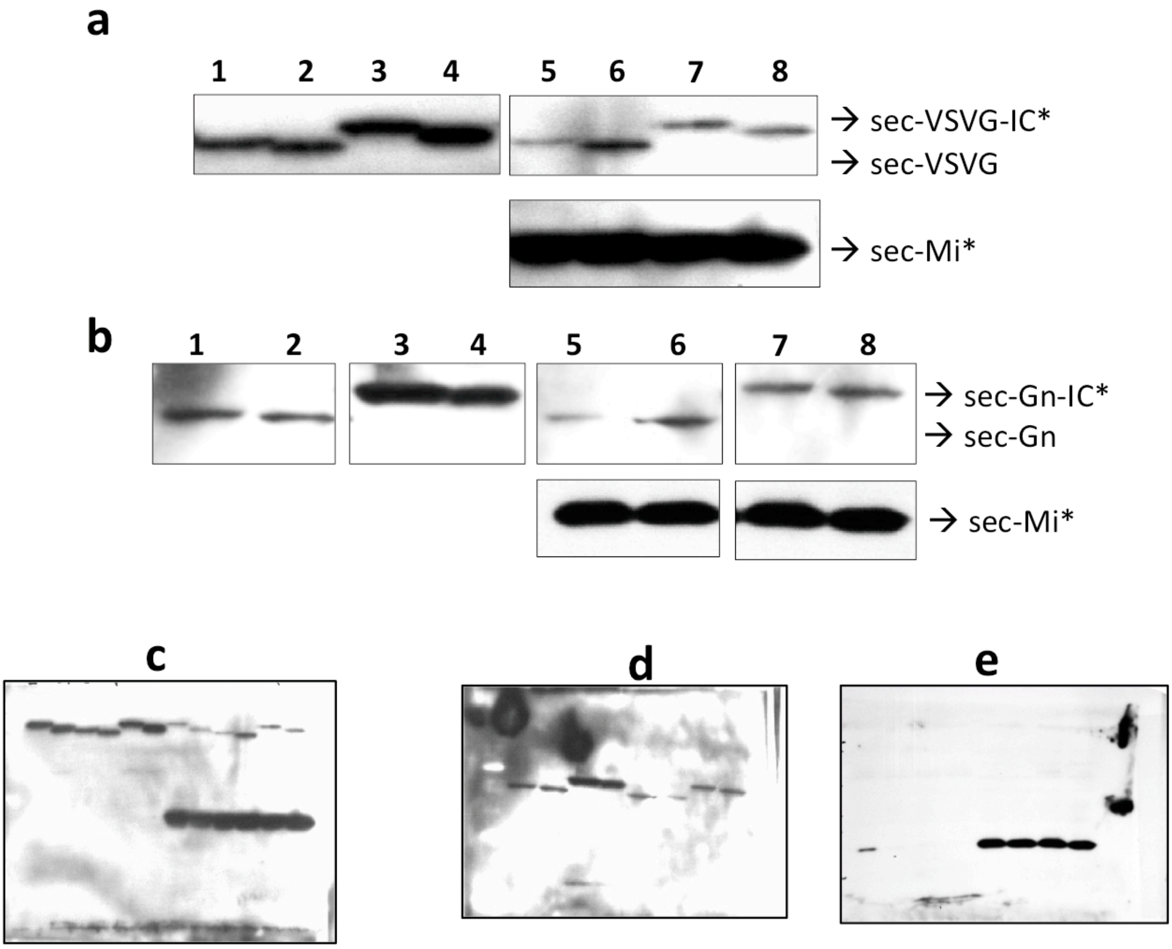

Figure S5: Glycosylation status of VSV-G and Gn glycoproteins. a. Composite figure made with Western blot shown in c- Western-blot analysis of DF-1 cells transfected (1-4) or co-transfected (5-8) with the expression plasmids for sec-SV5-VSV-G, sec-SV5-VSV-G-IC* (see Fig. S3 and Methods) and sec-muNS-Mi* (sec-Mi*). The proteins were treated (even numbers) or not (odd numbers) with N-glycosidase to remove the glycosylation. **b. Composite figure made with Western blots shown in d and e-** The same as in (a), but using sec-SV5-Gn and sec-SV5-Gn-IC* (see Fig. S3 and Methods) instead of the VSV-G constructs. The upper part (full length Western-blot shown in **d**) was revealed with anti-SV5 antibody, while anti-muNS antibody was used for the lower part (full length Western blot shown in **e**). Samples shown in lanes 5 and 6 were flipped because an error when loading the gel.

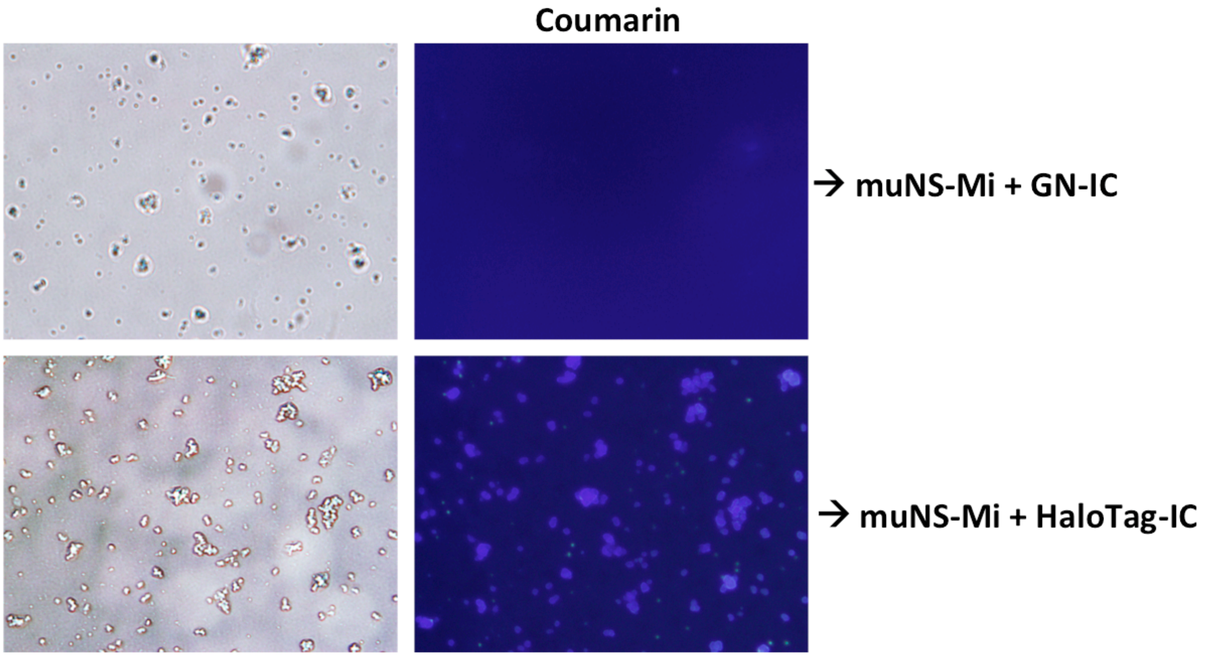

Figure S6: HaloTag-IC integrated in bacterially-produced MS retains enzymatic activity. Bacteria containing MS-integrated HaloTag-IC protein (see results) were disrupted, partially purified and incubated with 100 μM of coumarin-labelled HaloTag ligand for 20 minutes at room temperature. Then, samples were placed on a microscope slide and pictures were taken with a fluorescence microscope (lower pictures). MS containing Gn protein were used as a control (upper pictures). As shown, HaloTag integrated into MS are still able to specifically bind the HaloTag ligand.

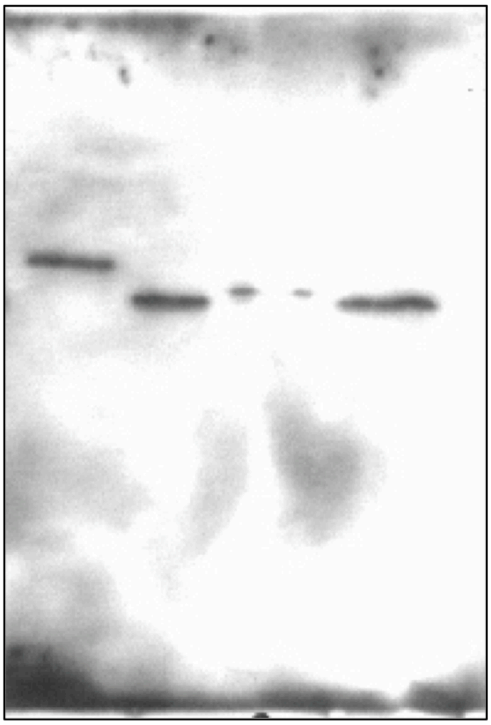

Figure S7: Full-length Western-blot corresponding to Figure 1d.

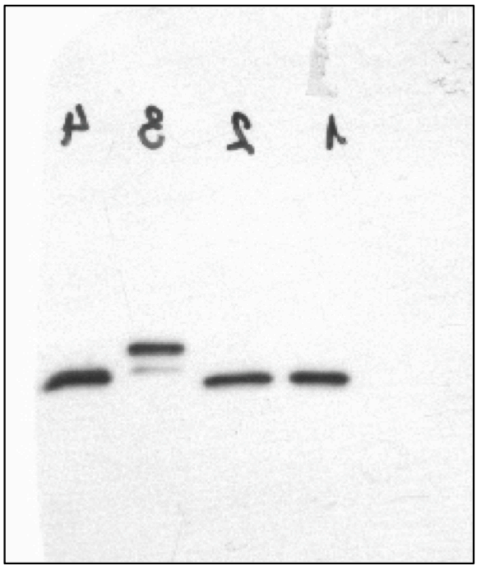

Figure S8: Full-length Western-blot corresponding to Figure 2a.

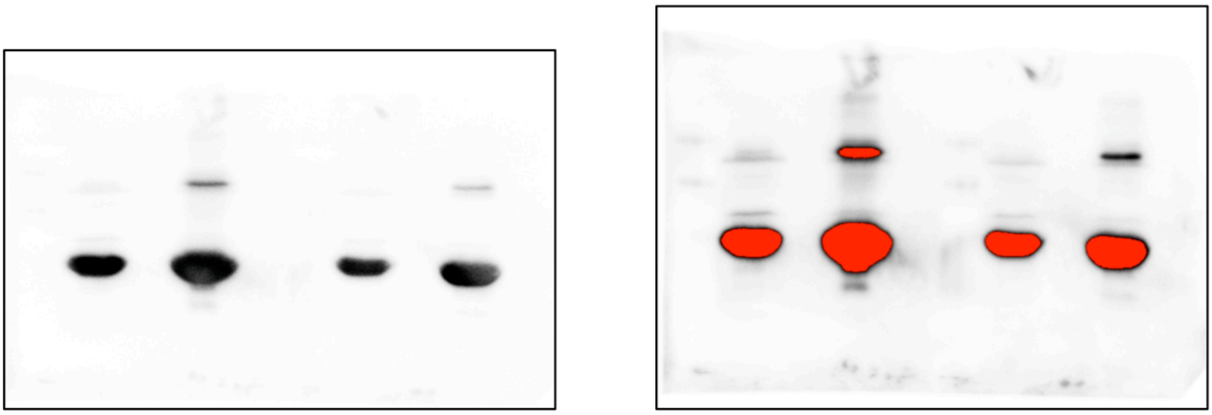

Figure S9: Full-length Western-blot corresponding to Figure 5b. Two different expositions are shown that were combined for Figure 5b.
